# Xylanase Production by Solid-State Fermentation for the Extraction of Xylooligosaccharides from Soybean Hulls^§^

**DOI:** 10.17113/ftb.61.04.23.8073

**Published:** 2023-12

**Authors:** Nataša Šekuljica, Sonja Jakovetić Tanasković, Jelena Mijalković, Milica Simović, Neda Pavlović, Nikola Đorđević, Alina Culetu, Ivana Gazikalović, Nevena Luković, Jelena Bakrač, Zorica Knežević-Jugović

**Affiliations:** 1 Innovation Center of Faculty of Technology and Metallurgy, Karnegijeva 4, 11000 Belgrade, Serbia; 2 University of Belgrade, Faculty of Technology and Metallurgy, Karnegijeva 4, 11000 Belgrade, Serbia; 3 National Institute of Research & Development for Food Bioresources-IBA, Ancuţa Băneasa 5, 021102 Bucharest, Romania

**Keywords:** soybean hulls, xylanase, *Penicillium rubens*, xylooligosaccharides, renewable materials, solid-state fermentation

## Abstract

**Research background:**

The development of a novel process for the production of xylooligosaccharides (XOS) based on the 4R concept is made possible by the integration of numerous techniques, especially enzymatic modification together with the physical pretreatment of renewable materials. This study aims to integrate the use of agricultural wastes for the production of xylanase by a new strain of *Penicillium* sp. and value-added products, XOS.

**Experimental approach:**

For the production of xylanase, a solid-state fermentation was performed using wheat bran as substrate. To obtain the most active crude extract of xylanase, the time frame of cultivation was first adjusted. Then, the downstream process for xylanase purification was developed by combining different membrane separation units with size exclusion chromatography. Further characterisation included determination of the optimal pH and temperature, determination of the molecular mass of the purified xylanase and analysis of kinetic parameters. Subsequently, the hydrolytic ability of the partially purified xylanase in the hydrolysis of alkali-extracted hemicellulose from soybean hulls was investigated.

**Results and conclusions:**

Our results show that *Penicillium rubens* produced extracellular xylanase at a yield of 21 U/g during solid-state fermentation. Using two ultrafiltration membranes of 10 and 3 kDa in combination with size exclusion chromatography, a yield of 49 % and 13-fold purification of xylanase was achieved. The purified xylanase (35 kDa) cleaved linear bonds β-(1→4) in beechwood xylan at a maximum rate of 0.64 μmol/(min·mg) and a Michaelis constant of 44 mg/mL. At pH=6 and 45 °C, the purified xylanase showed its maximum activity. The xylanase produced showed a high ability to hydrolyse the hemicellulose fraction isolated from soybean hulls, as confirmed by thin-layer chromatography. In the hydrothermally pretreated hemicellulose hydrolysate, the content of XOS with different degrees of polymerisation was detected, while in the non-pretreated hemicellulose hydrolysate, the content of xylotriose and glucose was confirmed.

**Novelty and scientific contribution:**

Future research focusing on the creation of new enzymatic pathways for use in processes to convert renewable materials into value-added products can draw on our findings.

## INTRODUCTION

The plant tissue contains a high concentration of biologically active components that are frequently used in pharmaceutical, cosmetic and food products. Biologically active components in plant tissues are stored in intracellular membranes and the extracellular matrix (*1*). Although the global industry offers a plethora of bio-based products and ingredients derived from plant biomass, oligosaccharides stand out due to all the beneficial effects attributed to them. Since oligosaccharides have all the properties of functional food supplements and are considered functional foods with therapeutic potential, the food industry is particularly interested in them (*2*). The fact that prebiotic oligosaccharides will reach a market value of $7.37 billion by 2023 shows that oligosaccharides have made the greatest scientific progress, which has boosted their commercial production (*2*,*3*). Nowadays, xylooligosaccharides (XOS), sugar oligomers consisting of xylose monomer units linked by β-(1→4) glycosidic bonds, are in particular focus (*3*). Due to their technological and nutritional properties, they are used in the food, pharmaceutical and agricultural industries, *e.g.* to improve dietary fibre content, viscosity colour intensity and to give food a firmer and stickier texture; as antioxidants, cholesterol-lowering and antihyperglycaemic agents in obesity, to balance the intestinal microbiota and for severe constipation in pharmaceutical applications; and in agriculture for dietary supplementation (*4*). Extraction of XOS from plant tissue is an extremely challenging and complicated multistep process because the structure of the plant cell is quite complex and consists of cellulose, hemicellulose and lignin fractions that are intertwined. Hemicellulose is the second most abundant polysaccharide whose basic building block is xylan, which consists of β-1,4-linked d-xylopyranose units in a linear framework with branched substituents such as arabinose, acetic acid, uronic acid, ferulic acid and coumaric acid observed at certain points in the structure (*5*). The conversion of xylan from agroindustrial residues to value-added XOS is considered a sustainable process. These processes for obtaining XOS are mainly two-step and consist of physical and chemical pretreatments of the lignocellulosic fraction aimed at reducing the complexity of the structure and partial solubilisation of xylan, and xylanase-catalysed hydrolysis of the resulting products to XOS with different degrees of polymerisation. The xylanase preparations can be added directly to the reaction, either immobilised or produced *in situ* by microorganisms (*4*,*6*).

The presence of different substituents and branches in the hemicellulose structure indicates that its hydrolysis and thus recovery of the XOS requires the simultaneous action of various enzymes such as endoxylanases and xylosidases together with arabinofuranosidases, ferulic acid esterases, uronidases and others (*5*). Xylanases are a class of hemicellulolytic enzymes that catalyse the degradation of xylan and convert plant biomass into products with commercial yield (*7*). They have gained popularity in the industry as they are used in a variety of applications, including saccharification of plant biomass, improvement of animal feed quality, production of food, juices and wine, and the natural sweetener xylitol (*5*). The use of xylanases in the saccharification and isolation of XOS from plant tissues requires the use of highly selective enzymes whose prices are prohibitive for industrial use. In this context, several studies have recently been conducted to investigate the lucrative production of xylanases using microorganisms, especially fungi, and waste biomass as substrates. The cultivation of fungi on solid substrate is a widely accepted approach for the production of a variety of industrially important enzymes, including xylanase, as this cultivation method is very similar to the conditions found in the natural habitats of fungi. For this reason, this type of cultivation provides a high yield of xylanase activity units when the composition of the nutrient substrate and the growth conditions are adequately controlled. Several fungal producers have been controlled to produce commercial xylanases. For example, Javed *et al.* (*8*) investigated the possibility of xylanase production in agricultural waste such as wheat bran using the fungus *A. niger* KIBGE-IB36. The xylanolytic potential of the endophytic fungus *F. graminearum* isolated from leaves of *Theobroma cacao* was investigated for the first time for the production of xylanase (low cellulase contents) when grown on wheat bran as the sole carbon source (*9*). *A. awamori* AFE1 and cassava peel were used for the production of thermally stable, acidophilic and surfactant-tolerant xylanase (*10*). In addition, xylanases were produced by solid-state fermentation of *A. niger* CCUG33991 using low-cost agro-industrial residues such as wheat bran, sorghum stover, corn cob and soybean meal in a tray bioreactor (*11*). *Penicillium crysogenum* was also used for the production of xylanolytic enzymes (*12*).

The aim of this work is to: (*i*) develop a method to use wheat bran as the sole carbon source for the production of xylanase with *Penicillium rubens*, (*ii*) develop a method to purify the produced xylanase, (*iii*) thoroughly biochemically characterise the partially purified xylanase and (*iv*) verify the activity of the partially purified xylanase in the biorefinery concept for valorisation of agricultural waste. For this purpose, the performance of the obtained enzyme was tested in the enzymatic extraction of XOS from the alkali-isolated hemicellulose from soybean hulls.

## MATERIALS AND METHODS

### Substrates and chemicals

Winter wheat of the Serbian variety Simonida was processed at a nearby mill in Čenta (Serbia) to obtain wheat bran containing on dry mass basis: protein (18.0±0.1) % (expressed as N×6.25), ash (3.9±0.1) % and cellulose crude fibre (6.9±0.1) % for cultivation of *P. rubens* in this study. The company Sojaprotein, Bečej, Serbia, provided soybean hulls for the production of xylooligosaccharides (XOS). Standard oligosaccharides for quantitative evaluation of soybean hull hydrolysates (xylose, xylobiose, xylotriose, xylotetraose and xylopentaose) were purchased from Megazyme (Wicklow, Ireland). Orcinol, agarose, arabinose, fructose, glucose, sucrose, Bradford reagent, bovine serum albumin, *n*-butanol, Sephadex G-75, gel filtration markers, glycerol and dithiothreitol (DTT) were purchased from Sigma-Aldrich, Merck (St. Louis, MO, USA). Beechwood xylan from Carl Roth (Karlsruhe, Germany) was used to test the xylanase activity. Bromophenol blue and Commasie Brilliant Blue R-250 were purchased from Thermo Fisher Scientific (Waltham, MA, USA). Sulfuric and acetic acid were purchased from Zorka Pharma (Šabac, Serbia), while aceton was purchased from Lach-Ner (Bratislava, Czech Republic).

### Screening and identification of xylanase producers

A competent producer of xylanases was selected from an extensive collection of fungal strains belonging to the Department of Biochemical Engineering and Biotechnology of the Faculty of Technology and Metallurgy, Belgrade, Serbia. The quick-DNA fungal/bacterial miniprep kit from Zymo Research (Irvine, CA, USA) was used to isolate genomic DNA from fungi. The isolated genomic DNA was then used for gene amplification by polymerase chain reaction (PCR). The internal transcribed spacer region (ITS) of the rRNA gene was amplified with a pair of universal primers (Ecogen, Barcelona, Spain), ITS1 (5'-TCCGTAGGTGAACCTGCGG-3') and ITS4 (5'-TCCTCCGCTTATTGATATGC -3'). PCR amplification was performed in 50 µL of the reaction mixture containing 1 µL DNA, 25 µL DreamTaq PCR Master Mix (Thermo Fisher Scientific), 0.5 µM each primer and nuclease-free PCR water supplemented to 50 µL. On the T100 thermal cycler Biorad™ (BIO-RAD, Hercules, CA, USA), PCR reactions were performed according to the following protocol: initial denaturation at 95 °C for 5 min, 30 cycles consisting of denaturation at 95 °C for 30 s, primer hybridisation at 55 °C (ITS) and 57 °C (28s rRNA) for 30 s, 72 °C extensions for 60 s and final extension at 72 °C for 7.5 min. Horizontal electrophoresis (20 min at 100 V) with 2 % agarose was used to determine the PCR product length. The PCR products were purified using the DNA Clean & Concentrator™ kit (Zymo Research) and sent to the sequencing service Macrogen (Amsterdam, The Netherlands) for sequencing. The sequences of the PCR products were analysed using the BLAST program (*13*) on the National Center for Biotechnology Information (NCBI) (*14*) website (www.ncbi.nlm.nih.gov/).

### Production of xylanase from P. rubens growning on wheat bran

Xylanase was prepared by solid-state fermentation (SSF). Fermentation was carried out in 100-mL Erlenmeyer flasks containing 5 g of wheat bran. The Erlenmeyer flasks filled with wheat bran were tightly sealed with cotton lids before autoclaving at 121 °C and 0.12 MPa for 20 min. The growth medium, *e.g.* wheat bran, was then moistened with 2 mL sterile citrate buffer (0.1 M, pH=5). The fungal isolate was grown on slanted malt agar (malt extract 20 g/L, agar 18 g/L) in an incubator at a temperature of 30 °C. After 72 h of incubation, the fungal isolate was scraped with 10 mL sterile distilled water containing a few drops of glycerol to prepare a spore suspension. Then, 1 mL spore suspension (10^6^ spore/mL) obtained by scraping the culture plate as described previously was aseptically added to the growth medium to perform inoculation. Xylanase was recovered from the fermented medium after 192 h of fermentation at 30 °C. A simple distilled water procedure was used to obtain xylanase from fermented wheat bran. Specifically, the fermented medium was mixed with 25 mL distilled water and incubated for 30 min at 30 °C and 150 rpm on an incubator shaker (KS 4000 I control; IKA, Staufen, Germany). The Heraeus^TM^ Fresco^TM^ 17 microcentrifuge (Thermo Fisher Scientific) was used at 16 800×*g* and 4 °C for 10 min to separate the crude extract containing xylanase, which was then stored at -20 °C until use. The obtained enzyme apparently retained its full activity after storage for 4 weeks.

### Analysis of enzymatic activities

The dinitrosalicylic acid (DNS) used to evaluate xylanase activity was described by Miller (*15*). The amount of reducing sugars released from xylan was measured spectrophotometrically at 540 nm using xylose as a standard. The unit of enzymatic activity (U) was defined as the amount of enzyme required to release 1 μmol xylose equivalent per minute under the assay conditions. Enzymatic activity was expressed as enzyme units per gram of substrate (U/g). Specific activities were expressed as enzyme units per milligram of protein (U/mg).

### Protein content determination

Protein concentration was determined by the Bradford method (*16*). Commercially available Bradford reagent was mixed with the sample according to the manufacturer's recommendations, and the protein content was calculated based on a standard curve prepared with bovine serum albumin (BSA).

### Purification of P. rubens xylanase

#### Ultrafiltration membrane fractionation

Ultrafiltration was used to initiate the purification of xylanase from *P. rubens*. Standard polyethylene sulfone membranes (Vivaflow^®^ 50; Sartorius AG, Göttingen, Germany; molecular mass cut-off 10 kDa) with a membrane area of 50 cm^2^ were loaded with crude xylanase extract using a peristaltic pump (Pumpdrive 5206; Heidolph, Schwabach, Germany) until a twentyfold volume reduction of the crude extract was achieved. After fractionation, protein content and xylanase activity were determined in permeate and retentate fractions. The xylanase-rich retentate was additionally concentrated using Amicon^®^ Ultra centrifugal filter units with a molecular mass cut-off of 3 kDa (Merck, Burlington, MA, USA).

#### Gel filtration chromatography

The xylanase-rich sample obtained after concentration using centrifugal filter units was chromatographed on a Sephadex G-75 (15 mm×250 mm, Omnifit^®^ Labware column; Sigma-Aldrich, Merck, St. Louis, MO, USA) pre-equilibrated with 100 mM citrate buffer, pH=6. The column was operated at a flow rate of 0.75 mL/min and the eluted fractions were collected using a fraction collector (Buchi C-660; Labortechnik AG, Flawil, Switzerland). The xylanase activity and protein content were determined in each fraction, as described above. While the other fractions in which xylanase activity was detected were pooled and freeze-dried (Beta 1-8 freeze-dryer; Martin Christ, GmbH, Osteroide am Harz, Germany), the fraction with the highest xylanase activity was kept for electrophoretic determination of xylanase purity and molecular mass. Gel filtration was used for molecular mass determination of purified xylanase. A mixture of gel filtration protein molecular mass markers, namely lysozyme from egg white (14.0 kDa), carbonic anhydrase from bovine erythrocytes (29.0 kDa), ovalbumin (44.0 kDa) and bovine serum albumin (66.0 kDa) were also separated on the same column under identical conditions and their elution volumes were determined. A standard plot was then made between *V*_e_/*V*_0_ (where *V*_0_ was initial and *V*_e_ elution volume in mL) on the x-axis and log *M* on the y-axis to calculate the molecular mass of the purified xylanase.

#### Sodium dodecyl sulphate–polyacrylamide gel electrophoresis

Electrophoresis was used to evaluate the efficiency of each step of xylanase purification. Sample buffers with the following composition: 2.5 mL of 2X Tris-HCl buffer, 2.0 mL glycerol, 2.0 mL of 1 M DTT solution, 4.0 mL of 10 % (*m*/*V*) sodium dodecyl sulphate (SDS) and 0.4 mL of 5 % (*m*/*V*) bromophenol blue were mixed with the samples collected at each stage of xylanase isolation at a volume fraction of 50 % and heated at 95 °C for 5 min. Samples were loaded onto a polyacrylamide gel (stacking: 4 % and resolving: 20 %) together with prestained protein ladder standard markers (10–260 kDa) and electrophoresis was performed at 120 V for 75 min in a vertical mini-Hoefer system (Hoefer, Holliston, MA, USA) equipped with an electrophoresis bath and a power supply. The gels were impregnated with Coomassie brilliant blue R-250.

### Purification parameters

The effectiveness of the purification process was evaluated by analysing the activity yield (*Y*/%) and purification factor. The following mathematical formulae were used to determine these parameters:


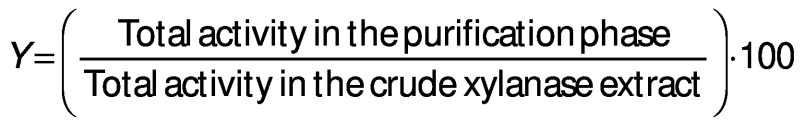
 /1/


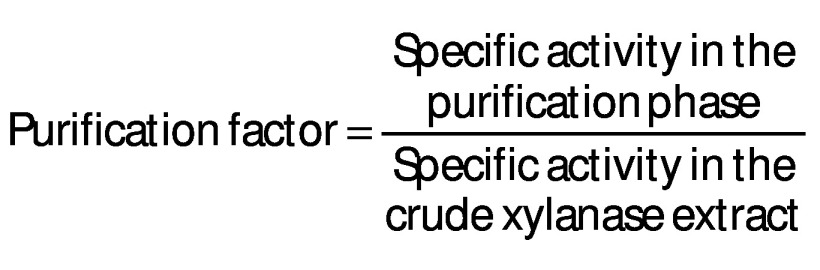
 /2/

### Characterisation of partially pure P. rubens xylanase

#### Effect of pH and temperature on the *P. rubens* xylanase activity

The optimal pH of partially pure xylanase was determined by measuring its activity in buffers with different pH values (3–10 in steps of 1 pH unit) such as sodium citrate buffer (100 mM, pH=3–6), sodium phosphate buffer (100 mM, pH=6–8), Tris-HCl (100 mM, pH=8–9) and glycine-NaOH buffer (100 mM, pH=10) at 37 °C. Enzyme activity was also measured at different temperatures (25, 37, 45, 50 and 60 °C) under standard assay conditions to determine the optimum temperature at pH=6.0.

#### Enzyme kinetics

In the evaluation of the kinetics of hydrolysis of beechwood xylan with *P. rubens* xylanase, the initial amount of the substrate was varied (0.25, 0.5, 1.0, 2.0, 3.0, 4.0 and 5.0 % (*m*/*V*)) and hydrolysis was carried out under optimal conditions for the indicated xylanase at pH=6 and *t*=45 °C. The conventional DNS method, as described previously, was used to follow the kinetics for each set of experimental conditions. The mathematical model of Michaelis-Menten kinetics included in OriginLab^®^ software package (OriginPro, v. 9.9) (*17*) was applied to the experimental data obtained. The degree of agreement between the experimentally obtained values and considered mathematical model was evaluated after applying the above mathematical model together with the values of kinetic constants *K*_m_ and *v*_max_.

### P. rubens xylanase-assisted production of XOS from soybean hulls

The hydrolysis of the hemicellulose of soybean hulls and the production of XOS with different degrees of polymerisation were performed with partially pure xylanase produced by *P. rubens*.

### Isolation of hemicellulose from soybean hulls for the production of XOS

Soybean hulls were pretreated with a mechanical mill (mixer mill MM 400; Retsch, Haan, Germany). In this process, 1 g substrate was ground into smaller particles with a size of 40–60 mesh using a standard ball for 120 s at 25 Hz. Subsequently, 10 g substrate powder were resuspended in 100 mL of 1.5 M NaOH and alkaline extraction of the hemicellulose fraction was performed as described (*18*). Briefly, the suspension was incubated at 80 °C for 60 min. The suspension was then cooled and the solid fraction was removed by centrifugation at 1870×*g* (Eppendorf^TM^ refrigerated centrifuge 5430 R; Thermo Fisher Scientific) for 10 min. The remaining supernatant (50 mL) was acidified to pH=5.5 with 6 M acetic acid and then cold ethanol (96 %, 150 mL) was added. After centrifugation at 1870×*g* for 10 min, the precipitate rich in hemicellulose was collected. The resulting precipitate was resuspended in distilled water and precipitated again. The extracted hemicellulose was freeze-dried for 24 h at a critical temperature of -40 °C in a laboratory freeze dryer (Beta 1-8 freeze dryer; Martin Christ, GmbH).

#### Enzymatic hydrolysis of extracted hemicellulose from soybean hulls

In this work, the enzymatic hydrolysis of extracted and hydrothermally pretreated hemicellulose with partially pure xylanase from *P. rubens* was studied under the previously defined optimal reaction conditions for specific xylanase. First, a suspension of 100 mg dried hemicellulose was prepared in 10 mL citrate buffer (0.1 M; pH=6). In addition, 100 mg dried hemicellulose were resuspended in 10 mL citrate buffer (0.1 M; pH=6) and incubated at 121 °C and 0.13 MPa for 1 h to achieve hydrothermal (HT) pretreatment (hot water and increased pressure) of the substrate. Then, the enzyme 10 U/g was added and both reactions were carried out at 45 °C for 5 h with constant mixing at 150 rpm (KS 4000 i control; IKA, Staufen, Germany). At the specified time intervals, samples were taken from the reaction mixtures and incubated at 100 °C (digital heating shaking drybath; Thermo Fisher Scientific) for 5 min to deactivate the enzyme, and the xylose concentration was determined by DNS assay.

#### Qualitative evaluation of soybean hull hemicellulose hydrolysates by thin layer chromatography

Thin layer chromatography with silica plates (5 cm×10 cm, Sigma–Aldrich, Merck) was used for qualitative characterisation of hydrolysates. The mobile phase for thin-layer chromatography (TLC) consisted of *V*(*n*-butanol):*V*(acetic acid):*V*(water)=2:1:1. The separation was performed in a TLC chamber (10.5 cm×12.5 cm) for 90 min. The plates were then dried in an oven at 90 °C and immersed in the staining solution consisting of 40 mL acetone, 2 mL concentrated sulfuric acid and 20 mg orcinol. After staining, the plates were dried at 90 °C for 5 min to support the development of the chromatogram.

#### Quantitative evaluation of soybean hull hemicellulose hydrolysates by high-performance liquid chromatography

Samples were analysed using Dionex Ultimate 3000, Thermo Fisher Scientific HPLC system. Analysis was performed using deionised water as a mobile phase with an elution rate of 0.6 mL/min on carbohydrate column (Hi-Plex Ca^2+^, 300 mm×7.7 mm, 8 µm; Agilent, Santa Clara, CA, USA) incubated at 80 °C. The sample injection volume was 20 μL and the analysis time was 25 min. The product was analysed using the RI detector (RefractoMax 520; ERC GmbH, Riemerling, Germany) preheated to 40 °C and the data acquisition and processing was done using Chromeleon v. 7.2 software (Thermo Fisher Scientific) (*19*). Analytical standards: glucose, fructose, sucrose, arabinose, xylose, xylobiose, xylotriose, xylotetraose and xylopentaose were used to generate calibration curves. Based on the obtained slope, the quantification of the target component in the obtained mixture was determined.

### Statistical analysis

All experiments in this study were performed in triplicate and the results are presented as mean value±standard deviation (S.D.). One-way analysis of variance (ANOVA) with Minitab^®^17 software (*20*) was used to compare the obtained results. The Tukey’s test was applied to compare the differences between the mean values at a confidence interval of 95 % (p<0.05).

## RESULTS AND DISCUSSION

### Kinetics of xylanase production

Xylanases are extremely important industrial enzymes and are estimated to have a large share of the global enzyme market (*21*). Therefore, a significant amount of xylanases with exceptional properties, such as enzyme activity and stability in difficult process environments, must be readily available for industrial use (*22*). Given the importance of xylanases, data are continuously collected on the microorganisms that produce them as well as on the growth conditions and composition of the culture medium in order to maximise the yield of xylanase enzymes. The Department of Biochemical Engineering and Biotechnology at the Faculty of Technology and Metallurgy, Belgrade, Serbia, has a large and diverse collection of fungal strains; therefore, the selection of a competent xylanase producer was considered the main task of this study. The first step in confirming the potential of the selected strain was the use of selective agar plates on which a xylanase enzyme activity index greater than 1.5 cm was observed (*23*). The strain was then identified by sequencing the ITS and NL genes. After amplification of the 28S ribosomal RNA region with a size of 600 bp and sequencing and comparison of the sequences with reference strains deposited in the NCBI database (*14*), the strain was found to belong to *Penicillium* spp. and was most likely *Penicillium rubens*. The identified strain was further used for the first time in the study of xylanase production by solid-state fermentation (SSF) and wheat bran as a substrate.

**Fig 1:**
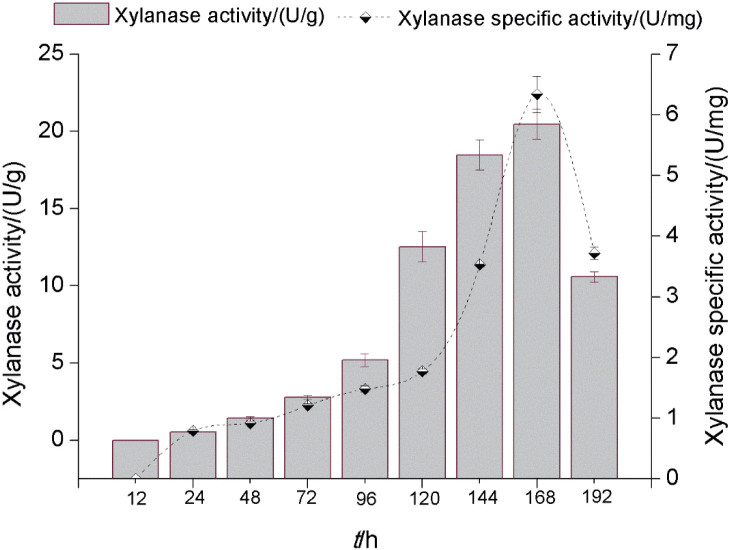
Kinetics of xylanase production by cultivation of *Penicillium rubens* on wheat bran under the regime of solid-state fermentation (fermentation conditions: temperature=30 °C, pH=5.0), each point represents the mean value±S.D., *N*=3

The best inducer of xylanase synthesis is xylan. However, due to the prohibitive price of xylan, the use of pure xylan as a carbon source for the production of xylanases is considered unattractive. Therefore, it has been recognised that using agricultural waste as the sole carbon source for xylanase synthesis could be a good solution. Although wheat bran is considered a waste, there is strong evidence that the bran is recognised as a suitable substrate for xylanase production because of its hemicellulosic nature (presence of nonstarch polysaccharides, 41 to 60 %), favourable degradability and the presence of some nutrients (*24**,**25*). Furthermore, the biochemical composition shows that after hydrolysis of wheat bran, significant amounts on dry mass basis of soluble sugars such as glucose (42.5 %), xylose (15.4 %), arabinose (3.1 %) and galactose (2.7 %) are produced, which are necessary to initiate the growth of microorganism and promote their further growth (*26*). Wheat bran xylans consist of β-d-(1,4)-linked xylopyranosyl skeleton and can be replaced by α-l-arabinofuranosyl as the main group of pendants for O_2_ and/or O_3_, making it an appropriate source for the production of xylanase (*27*). For this purpose, *P. rubens* was incubated for 192 h on wheat bran at 30 °C and harvested regularly and the data on the activity of xylanase in crude extracts were collected and shown in Fig. 1.

With a few exceptions, xylanase production by filamentous fungi occurs in cultures with an initial pH<7.0 (*28*). Under these fermentation conditions, the xylanase activity titre in the extract increases with increasing fermentation time until it reaches its peak after 168 h, as shown in Fig. 1. Thus, the maximum xylanase production with *P. rube*ns was measured after 168 h of fermentation and reached a value of 21 U/g of substrate. These results indicate that wheat bran is a suitable substrate for the cultivation of *P. rubens* and the production of xylanase because in addition to cellulose, it contains hemicellulose in high concentrations, which is also recognised as an inducer of xylanase production (*29*). In addition to cellulose and hemicellulose, xylanase synthesis on wheat bran is thought to be significantly affected by unquantified specific trace elements, including minerals (*30*). Fig. 1 shows a strong increase in xylanase activity from 12 to 168 h, particularly from 96 to 168 h of fermentation, in addition to a strong decrease in the activity after 168 hours of fermentation. The decrease in the amount of inducer taken up during the earlier stages of microorganism growth could be the cause of this decrease in activity. In addition, overexposure of lignin through the consumption of xylan increases the likelihood of the occurrence of phenolic compounds that inhibit xylanase activity (*30*).

The production of xylanases by various fungal strains using wheat bran as a carbon source in fermentation on a solid substrate is well documented in the literature. *Penicillium echinulatum*, for example, produced 10 U/g of xylanase under solid-state conditions with wheat bran (*29*). When *A. niger* XY-1 was cultured under SSF conditions in the presence of wheat bran, it showed remarkable xylanase production values. The xylanase activity on dry substrate basis reached 14.64 U/g after 48 h of fermentation (*31*). After optimisation of the process parameters, wheat bran stimulated high xylanase secretion (on dry carbon source 7.83 U/g) by *H. lanuginosa* in SSF (*32*). Although wheat bran proved to be an excellent substrate for xylanase production, further investigations and data analyses revealed that slight enrichment of the substrate had a significant effect on the increase in xylanase yield. The enrichment of wheat bran with a modified inorganic Czapex-Dox medium is one such example. More specifically, wheat bran moistened with a modified inorganic Czapex-Dox medium was used for the production of xylanase with the strain *Rhizopus oryzae* SN5. The maximum yield of xylanase, 273.83 U/g, was achieved at 30 °C and pH=6.0 after 5 days of incubation (*33*). In addition, *A. niger* NFCCI 4113 was used to produce xylanase with wheat bran as a carbon source and yielded 771.37 U/g after 144 h of fermentation at 30 °C, pH=5 and an initial moisture content of 75 % (*34*). Similarly, Dobrev *et al.* (*35*) observed that the use of wheat bran as a substrate in SSF resulted in good xylanase production. Wheat bran offers several possibilities for combination with other substrates to increase the yield of the target enzyme, in this case xylanase, and can also be used as the sole carbon source for microbial growth during fermentation. Since the selected strain *P. rubens* and the substrate wheat bran have been shown to be suitable for the production of xylanase, one of the research directions could be the optimisation of the parameters of fermentation together with the use of a consortium of microorganisms, which would increase the use of the substrate and thus a better yield of the activity of the enzyme units can be expected (*36*).

### Partial purification of xylanase

The potential environmental impact of enormous waste streams in the extraction of enzymes from complex fermentation media has already been greatly minimised by choosing fermentation on solid-state instead of submerged fermentation for xylanase production. Precipitation with ammonium sulfate or organic solvents is often used as a first step in enzyme purification processes from complex fermentation media. However, if we want to produce enzymes on an industrial scale, we need to consider the purification steps, or more precisely, we need to reduce the consumption of chemicals. Therefore, in this study, the xylanase produced by culturing the new fungal strain on wheat bran was partially purified using membrane separation techniques and column chromatography. In the first stage of the purification protocol, membranes with a pore size of 10 kDa were used to concentrate the crude xylanase extract obtained. In this way, two streams, retentate and permeate, are separated. The presence of xylanase in the retentate was detected by a DNS activity assay, which indicated that the molecular mass was above 10 kDa. From Table 1, which summarises the purification of xylanase, it can be deduced that the chosen membrane separation procedure can be considered as the first stage of purification of the produced xylanase. This is demonstrated by the fact that only 5 % of the activity of the preparation was lost after the first purification step, although the purity of the preparation was increased fourfold. In comparison, the use of ammonium sulfate in the first step of xylanase purification from *A. fumigatus* resulted in an activity yield of 51 %, demonstrating that the membrane technique is superior to the use of ammonium sulfate in the first steps of xylanase purification (*31*). To simplify chromatography and obtain representative results, the prepared xylanase preparation was then highly concentrated using centrifugal filters (3 kDa) and chromatographed on a Sephadex G-100 gel column.

**Table 1:** Purification of xylanase from *Penicillium rubens*

Purification step	A/(U/mL)	A_total_/U	*Y*/%	*γ*(protein)/(mg/mL)	PF
Crude extract	1.05±0.01	195.3±1.2	100.0±0.0	7.7±0.4	1.0±0.0
Membrane, 10 kDa	16.9±1.0	185.5±3.2	95.0±2.3	26.0±1.3	4.8±0.1
Membrane, 3 kDa	61.4±1.2	128.9±2.2	66.0±2.3	84.6±2.1	5.3±0.2
SEC, 100 kDa	9.1±0.6	95.3±1.4	48.8±1.6	5.2±0.2	12.9±0.6

A=xylanase activity, *Y*=yield, PF=purification factor, SEC=size exclusion chromatography

**Fig. 2:**
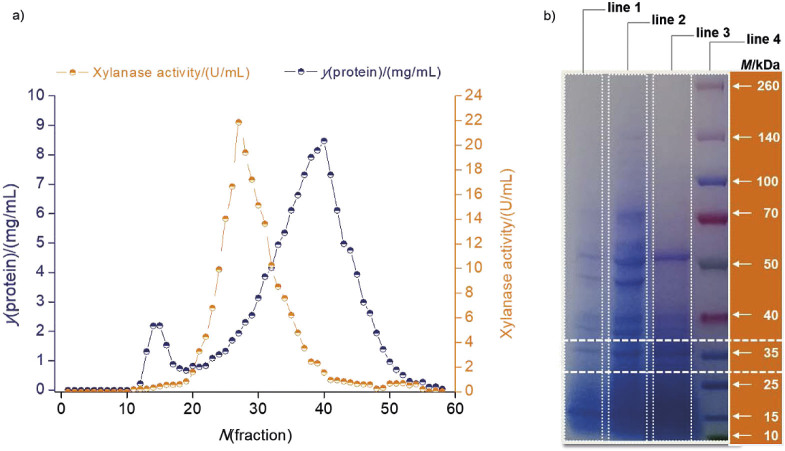
Results of chromatographic and electrophoretic analyses: a) size elution profile of xylanase and b) electrophoretic representation of the various steps in the purification of xylanase on a 4 % stacking and 20 % resolving polyacrylamide gel: line 1=crude xylanase extract, line 2=partially purified xylanase using a membrane with molecular mass cut-off 10 kDa, line 3=a fraction with maximal xylanase activity collected from size exclusion column and line 4=molecular mass markers

Fig. 2 shows the chromatogram of partially purified xylanase sample obtained after using membrane separation units, which indicates two peaks. By studying the xylanase activity and protein concentration of each fraction, it was determined which fractions contained xylanase. The activity study showed that xylanase was in fractions *N*=20 to *N*=40.

Gel filtration chromatography was used for the estimation of the molecular mass of partially purified xylanase. Based on the elution volumes of the enzyme and standard markers, the molecular mass of xylanase was calculated to be approx. 36 kDa. By observing the electrophoretic mobility (Fig. 2a) and the positioning of the bands in all samples obtained in all purification steps, this estimate was confirmed. The molecular mass of partially purified xylanase was determined to be 35 kDa based on the collected fractions and the activity and position of the bands in the electropherogram. The molecular sizes of xylanases isolated from fungi ranged from negligible to very large; however, the results showed good agreement with the molecular sizes of xylanases isolated from *A. oryzae* and *Beauveria bassiana* (37 kDa) (*37*,*38*). The fractions in which xylanase activity was detected were pooled, freeze-dried and used for further enzyme characterisation.

### Biochemical characterisation of partially pure xylanase

**Fig. 3:**
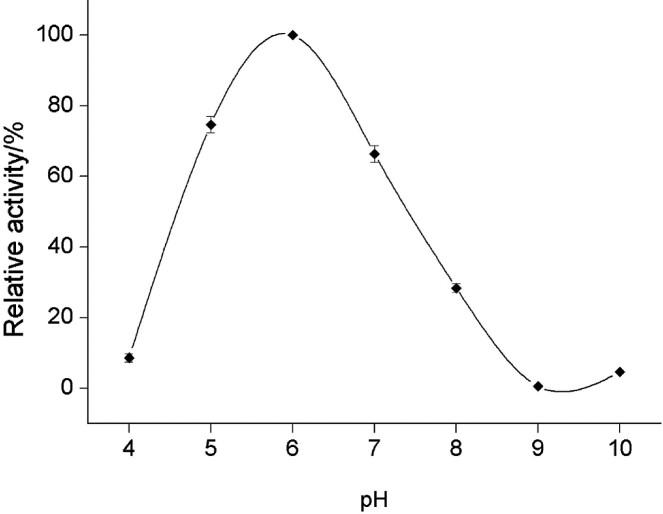
Effect of pH on the activity of *Penicillium rubens* xylanase. Each point represents the mean value±S.D., *N*=3

The success of enzyme-catalysed reaction determines the charge on the substrate and the arrangement of charged groups on the enzyme. In this context, it is necessary to investigate how pH affects a particular reaction. The effect of pH on partially pure xylanase is shown in Fig. 3. The xylanase activity of *P. rubens* was highest at pH=6 (Fig. 3) but xylanase also showed significant activity at pH=5 and 7, reaching 70 % in this pH range. As the enzyme retains less than 20 % of its activity at pH values above 8 and below 5, these conditions are not acceptable for the xylanase tested. The results of this study are fully consistent with previously collected data on the pH ranges in which fungal-derived xylanases function best. There are a few xylanases that prefer an alkaline environment, but the vast majority of fungal-derived xylanases are acidophilic (*37*).

**Fig. 4:**
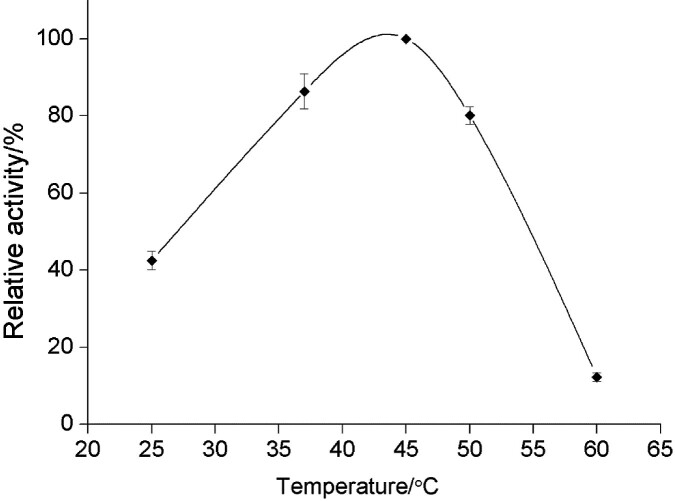
Effect of temperature on the activity of *Penicillium rubens* xylanase. Each point represents the mean value±S.D., *N*=3

The temperature optimum of partially pure xylanase from *P. rubens* was determined by a conventional hydrolysis reaction of xylan from beechwood at different temperatures and the influence of temperature on xylanase activity is shown in Fig. 4. A significant increase in xylanase activity was observed at temperatures between 25 and 45 °C (Fig. 4). Xylanase optimum activity was observed at 45 °C. However, it is also very important to emphasise that a high percentage of activity was maintained at temperatures above 45 °C. Xylanase was found to retain 80 % of its activity at 50 °C. Further increasing the reaction temperature above 50 °C is not beneficial for the selected xylanase as a significant decrease in the activity was observed under these conditions.

Similar results were found in other studies on the biochemical characterisation of xylanase from *Penicillium* strains: *P. purpurogenum* (pH=7.0, 50 °C) (*39*), *P. sclerotiorum* (pH=4.5, 50 °C) (*40*) and *P. ocitanis* (50 °C) (*41*). In summary, stable xylanases are desirable for various industrial processes, especially those requiring extreme conditions.

### Kinetic analysis of partially purified P. rubens xylanase

The kinetic properties of partially pure xylanase were studied by changing the initial concentration of the substrate xylan from beechwood and modelling the experimental data with a mathematical model reflecting Michaelis–Menten kinetics. The data obtained show that the enzyme obeys Michaelis–Menten kinetics (*R*^2^=0.9930). Here, the kinetic parameters for xylanase produced by *P. rubens* were calculated and it was found that *K*_m_ and *v*_max_ for beechwood xylan were 43.74 mg/mL and 0.64 μmol/(min·mg), respectively. These values are consistent with the kinetic parameter values of other fungal xylanases, which range from 0.09 to 40.9 mg/mL for *K*_m_, depending on the substrate used. The data obtained in this study show that the partially pure xylanase has a high affinity for the substrate, which is important for the industrial use of the enzyme (*42*).

### Production of XOS from soybean hulls using partially purified P. rubens xylanase

**Fig. 5:**
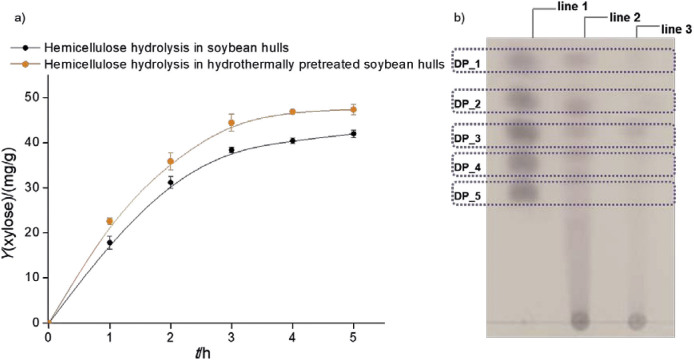
Results for: a) time course of the hydrolysis of hemicellulose from soybean hull catalysed by partially purified xylanase from *Penicillium rubens* (reaction conditions: temperature=45 °C, pH=6.0, Xylanase activity=10 U/g, initial substrate content=1 % *m*/*V*) and b) thin-layer chromatography of hydrolysis products: line 1=standard XOS markers (xylose, xylobiose, xylotriose, xylotetraose and xylopentaose), line 2=hydrolysate of hydrothermally pretreated soybean hull and line 3=soybean hull hydrolysate. Each point represents the mean value±S.D., *N*=3

Data are constantly being collected on the efficiency of the use of xylanase in the conversion of lignocellulosic feedstocks as this process alleviates the problem of agricultural waste while providing numerous industrially important products. Documented data on the production of XOS from lignocellulosic materials mainly refer to techniques using commercial xylanases. Therefore, this study investigated the feasibility of using partially pure xylanase from *P. rubens* in the hydrolysis of the hemicellulose fraction from soybean hulls extracted by alkaline treatment. In addition, the separated hemicellulose fraction was subjected to high-temperature and pressurised water treatment, known as HT treatment, to determine whether pretreatment prior to enzymatic hydrolysis was necessary to maintain or increase the yield of XOS. The DNS method was used to follow the progress of the hydrolysis reaction and the results are expressed as xylose equivalents (Fig. 5a). A qualitative evaluation of the resulting hydrolysate was then performed by thin-layer chromatography (Fig. 5b) to determine the presence of the XOS and the degree of polymerisation (DP). The sugar content was then determined by HPLC (Table 2).

**Table 2:** A summary of the yield of xylooligosaccharides (XOS) on the initial biomass basis after enzymatic hydrolysis

**Monosaccharide**	**Hydrolysis strategy**	
Without HT pretreatment	With HT pretreatment
*Y*(monosaccharide)/(mg/g)	
**Arabinose**	nd	1.88±0.05
**Glucose**	6.5±0.4	6.70±0.30
**Xylose**	nd	2.02±0.03
**XOS**	*Y*(XOS)/(mg/g)	
**Xylobiose**	nd	4.29±0.08
**Xylotriose**	10.7±0.3	2.49±0.05
**Xyotetraose**	nd	2.20±0.20
**Xylopentaose**	nd	1.69±0.02

nd=not detected

As can be seen in Fig. 5, the content of reducing sugars expressed as xylose equivalents increases during the hydrolysis of both untreated and HT treated hemicellulose, indicating the affinity of the partially purified xylanase for the selected substrate. More precisely, in the hydrolysate obtained with pretreated hemicellulose from soybean hulls a significant increase (p<0.05) in reducing sugar mass fraction was observed ((47.4±1.2) mg/g) compared to the mass fraction of reducing sugars in hydrolysate obtained by hydrolysis of non-pretreated hemicellulose from soybean hulls ((42.0±0.8) mg/g), indicating that the hydrolysis with pretreatment is preferable in terms of the output of total reducing sugars. The reported data are consistent with previously reported data on the effects of HT treatment on the extractability of XOS (*6*). Indeed, the HT treatment led to solubilisation and fragmentation of xylan into soluble xylooligomers, which were converted by xylanase into functional XOS with different degrees of polymerisation (*6*). Comparing the yield of XOS obtained by two different routes, the process using non-pretreated hemicellulose for XOS production yielded up to (10.6±0.4) mg/g, while HT treatment resulted in an almost equal yield of (10.5±0.3) mg/g. Although the XOS content in both hydrolysates was not significantly different (p<0.05), the XOS profile differed significantly (Table 2).

The profile of reducing sugars in the hydrolysate obtained by hydrolysis of the non-pretreated hemicellulose is characterised by the dominant mass fraction of glucose ((6.5±0.4) mg/g) and xylotriose ((11.0±0.1) mg/g). On the other hand, the hydrolysate obtained by the hydrolysis of hemicellulose previously treated with hot water under high pressure was rich in xylobiose, xylotriose, xylotetraose and xylopentaose with the highest mass fraction of short XOS, xylobiose ((4.3±0.1) mg/g). The combination of hot water and high pressure destroys hemiacetal bonds in hemicellulose, thus promoting the extraction of oligosaccharides from hemicellulose or, more specifically, enzymatic hydrolysis (*39*). After deacetylation, specific regions in the hemicellulose structure become accessible to the enzyme, which is reflected in the change of the XOS profile in the hydrolysate (*6*). In addition, arabinose was detected in the hydrolysate obtained after HT treatment, which was not present in the hydrolysate of the hemicellulose that had not been hydrothermally pretreated. It seems that the partially pure xylanase preparation, which in addition to xylanase also contains α-l-arabinofuranosidase and β-xylosidase (data not shown), contributes to the saccharification of the hemicellulose fraction of soybean hulls, as evidenced by an increase in the yield and type of monosaccharides such as arabinose and xylose, which have been confirmed to be antiglycaemic agents and to have an exceptional effect on human health (*41*). From these results, it can be concluded that the observed process justifies HT treatment prior to enzymatic hydrolysis.

## CONCLUSIONS

Xylanase was produced by solid-state fermentation of wheat bran with *Penicillium rubens*. A purification process based on membrane separation and column chromatography was developed for the produced enzyme, reducing the use of chemicals and environmental impact. The enzyme showed optimal activity in a pH range of 5 to 7 and a temperature range of 37 to 50 °C. The hydrolysates produced with different strategies resulted in xylooligosaccharides (XOS) with different degrees of polymerisation, which was confirmed by thin-layer and HPLC chromatography. Based on the obtained results, hydrolysates containing XOS can be considered as promising mixtures as they contain a high percentage of xylobiose, which is known as a prebiotic. The use of a new xylanase with improved catalytic properties over lignocellulosic substrates could be a viable technique to increase process yield and efficiency and is therefore ideal for inclusion in a global biorefinery plan for soybean hull use.
